# From animal models to new approach methodologies: Opportunities and challenges

**DOI:** 10.1016/j.xinn.2026.101260

**Published:** 2026-01-06

**Authors:** Haiying Zhu, Xinyu Lin, Qinjie Weng, Ji Cao, Qiaojun He, Bo Yang

**Affiliations:** 1 School of Medicine, Hangzhou City University, Hangzhou, China; 2 Center for Drug Safety Evaluation and Research, College of Pharmaceutical Sciences, Zhejiang University, Hangzhou 310058, China; 3 Institute of Pharmacology and Toxicology, Zhejiang Province Key Laboratory of Anti-Cancer Drug Research, College of Pharmaceutical Sciences, Zhejiang University, Hangzhou, China; 4 The Innovation Institute for Artificial Intelligence in Medicine, Zhejiang University, Hangzhou, China

## Abstract

Animal testing has long served as a foundational aspect of drug development. Since the US Food and Drug Administration (FDA)’s April announcement to phase out mandatory animal testing, health and regulatory agencies worldwide—including the NIH, European Commission, European Medicines Agency, and the United Kingdom’s Health and Safety Executive—have signaled efforts to promote new approach methodologies (NAMs) and reduce reliance on animal testing. Although NAMs have developed rapidly in recent years, there is considerable debate regarding whether they can truly replace animal experiments. Here, we provide a comprehensive analysis of the latest research comparing the strengths and limitations of NAMs with those of traditional animal testing while exploring potential paradigm shifts in global drug development strategies.

## Transition to new approach methodologies

The current scientific consensus emphasizes the urgent need to accelerate the development of alternative models and technological approaches that more accurately reflect the authentic human biology. Meanwhile, the US Food and Drug Administration (FDA) explicitly encourages the adoption of organoids and microphysiological systems (MPSs) to enhance drug safety and reduce reliance on animal models (Figure 1).

**Figure 1:**
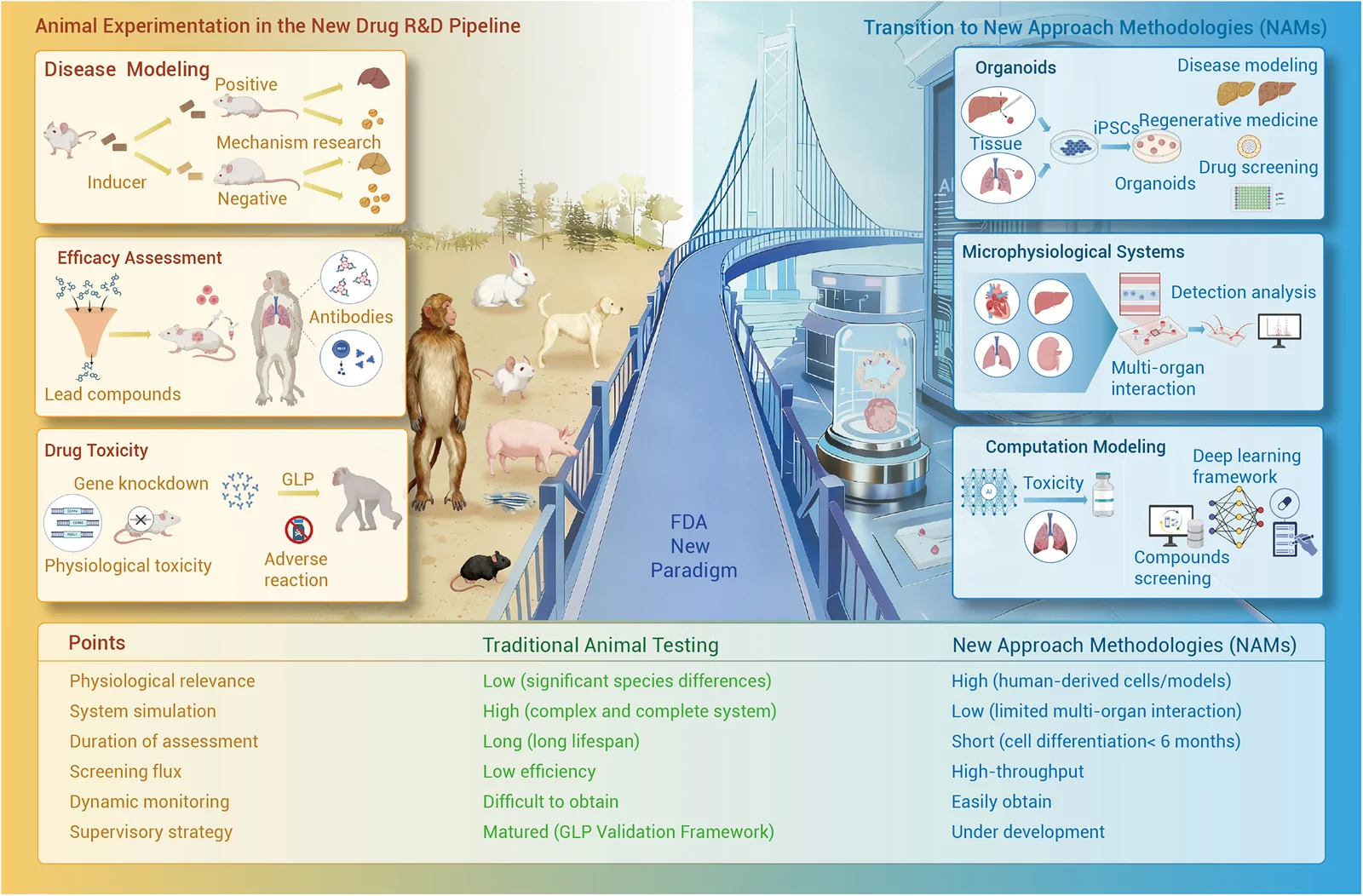
New approach methodologies are gradually replacing animal experiments in drug development

### Organoids for efficient drug screening

Organoids are advanced *in vitro* 3D human microtissue models that replicate organogenesis, disease progression, and individualized responses, providing a transformative alternative to animal models. For instance, only 35.3% of genes expressed in the human liver are shared with mice, which requires human-based systems to model development and disease. Earlier multicellular liver organoid models lacked key features such as functional bile ducts and physiological liver zonation. In recent years, increasingly complex and physiologically precise organoid models have been continuously developed. Hasan et al. developed highly structured, self-assembling hepatic organoids derived from induced pluripotent stem cell (iPSC)-derived liver progenitor cells within optimized culture systems. These organoids successfully replicate multi-zonal liver development and disease pathology, providing a physiologically relevant, humanized platform for drug screening.^1^ Furthermore, recent advances have been achieved in modeling the significantly greater complexity of the liver system. Research led by Wu et al. recently developed a methodology for differentiating human pluripotent stem cells (hPSCs) into vascularized cardiac and hepatic organoids, directly evaluating pharmaceutical effects on human liver development while significantly improving liver vascularization techniques.^2^ Similarly, researchers have generated adult liver cell organoids with metabolic functions, capable of lipid elimination following treatment with compounds implicated in metabolic dysfunction-associated fatty liver disease.

Beyond hepatic models, organoid technology extends to a wide range of applications. For instance, human gastroids have been developed to model gastric fundic-antral patterning *in vitro*, and study reveals that multilayered co-development depends on non-endodermal cells, particularly neural clusters.^3^ Methodologies for generating fetal epithelial organoids from amniotic fluid cells may overcome long-standing ethical and legal barriers in fetal tissue research. These organoid systems, with high physiological fidelity and translational potential, are reshaping research from fundamental studies to personalized therapies.

### MPSs for multi-organ research

MPSs, also known as “organ-on-a-chip” platforms, emulate the complexity of human tissues, permitting the investigation of organ-level responses to pharmaceuticals, toxins, and disease processes. The organ-on-a-chip platform developed by Wyss Institute at Harvard University, a pioneer in MPSs, has created a viable human body-on-chips (HuBoC) system, achieving interconnection among eight different organ modules, including the heart, liver, lungs, and kidneys.^4^ However, each module contains about one millionth of the number of cells in actual human organs, which may reduce sensitivity for the detection of low-abundance biomolecules. Recently, researchers further combined organoids with organ-on-a-chip technology to create organoid-on-a-chip systems, which enhance three-dimensional complexity and permit real-time monitoring. For instance, researchers developed an organ-on-a-chip system integrating human CD14^+^ monocytes and mature heart, liver, bone, and skin tissues via recirculating vascular flow. It evaluates doxorubicin’s multi-organ toxicity and establishes a clinical pharmacokinetics and pharmacodynamics (PK/PD) profile.^5^ Overall, organ-on-a-chip systems are more sensitive than animal testing. A recent study showed that the emulate chip identified drug-induced liver injury (DILI)-causing compounds and non-DILI-causing compounds with 87% and 100% accuracy, respectively, whereas such toxicity often goes undetected in animal experiments.^6^ These multicellular engineered systems provide practical avenues for the pharmaceutical industry to reduce dependence on animal models and adopt more human-relevant preclinical testing strategies.

### Computational modeling predicts drug toxicity and efficacy

Computational modeling and simulation utilize computer algorithms and mathematical frameworks to recreate biological processes, drug interactions, and disease progression. The first small-molecule drug, rentosertib, identified under the guidance of artificial intelligence (AI), has entered phase 2a trials and demonstrated good safety and tolerability in the treatment of idiopathic pulmonary fibrosis.^7^ Collins et al. have recently developed an interpretable deep learning framework and successfully screened out antibiotics with novel structural scaffolds from a pool of over 12 million compounds.^8^ Similarly, an AI tool developed by Hartung’s laboratory predicts compound performance in nine traditional animal tests, such as eye irritation and genetic mutation, with over 80% agreement, surpassing the reproducibility of animal tests themselves.^9^ In an era when personalized immunotherapy is progressively replacing cytotoxic chemotherapy as the mainstream systemic cancer treatment, it is essential to further integrate real-world data with traditional clinical and demographic variables to improve target identification and predictive accuracy prior to treatment initiation.

## Are traditional animal models essential in drug development?

Animal models continue to serve as a pivotal instrument in preclinical drug testing. Humanized mouse models, created by transplanting human cells or tissues or through genetic engineering, serve as essential platforms for drug development and immune disease studies. The development of biological agents, such as antibodies and gene therapies, relies heavily on primate toxicological data due to the high degree of immunological homology with humans. At the onset of the COVID-19 pandemic, rhesus and crab-eating macaques generated crucial virological data and supported efficacy and toxicity evaluation of mRNA and viral vector vaccines. Together, these approaches underscore the continuing importance of animal-based assessments in clarifying pharmacological mechanisms, quantifying therapeutic effects, and predicting human clinical efficacy.

While regulatory shifts toward non-animal methods represent progress in drug development, fundamental biological research still relies on animal models due to several limitations. (1) Pathophysiological research limitations: NAMs are useful for refining drug efficacy testing but cannot fully replace animal models in studying complex pathophysiology. For example, organoids and organ-on-chip technologies struggle to model whole-organism immune responses such as cytokine storms or pathogen dissemination across the blood-brain barrier. (2) Risks in pandemic response: a shift toward NAMs may compromise rapid response capabilities during novel pandemics, as standardized organoid models take 3–6 months to develop. AI-driven models depend on pre-existing datasets and are consequently limited in their predictive capacity during emerging outbreaks involving novel pathogens, where relevant data are unavailable. (3) Drug toxicity concerns: high-dose toxicology studies in animals are indispensable for setting initial human dosing parameters and identifying off-target organ toxicities. Emerging technologies enhance secondary screening processes but lack the fidelity and regulatory validation necessary to supplant whole-animal studies for primary toxicological risk assessment. (4) Divergent organoid standards: organoids show variability in metabolic enzyme activities due to differences in stem cell sources and culture media, impacting toxicity test reproducibility. Although the FDA plans to establish a NAM database for standardization, agencies such as the EMA, PMDA, and China NMPA prioritize different validation frameworks, hindering cross-border data harmonization. Without global alignment, conflicting interpretations of NAM-generated toxicology data may prolong drug approval timelines. Complementary animal models remain essential during transitions.

## Outlook: New paradigm of NAM-driven biopharma

As early as 1985, the FDA began promoting *in vitro* tests and, in April 2025, decided to phase out mandatory animal testing for certain therapeutics, including monoclonal antibodies and other pharmaceuticals. This policy transition, grounded in the long-standing “3 R” principles (replacement, reduction, and refinement), is driven by several key factors: persistent ethical concerns regarding animal experimentation, recognized limitations in the translational predictivity of animal models for human outcomes, and the growing sophistication and validation of alternative NAMs. These technologies offer higher physiological relevance and enable faster, cost-effective evaluation of chemical toxicity and efficacy.

On the one hand, *in vitro* experimental models are essential tools for drug evaluation. For example, human-specific antigens (such as the Gp100 protein) do not have corresponding targets in animal models, making *in vitro* approaches more appropriate for assessing efficacy and safety. Furthermore, advanced models are necessary to retrospectively elucidate the mechanisms of toxicity underlying unpredictable clinical events that were not identified in animal studies. On the other hand, the new policy pressures the biopharmaceutical sector across technological, scientific, and industrial fronts, setting a global benchmark for next-generation drug evaluation. Resources will shift toward FDA-compliant technologies, influencing the pace and direction of global biomedical progress.

Pioneering regulatory integration: it will be necessary to establish international, multicenter clinical data-sharing platforms based on real-world evidence. Such advancement necessitates the rapid development of harmonized frameworks to facilitate global data reciprocity and regulatory standardization. Furthermore, we advocate for the establishment of purpose-built data repositories to enhance the quality and scope of data collection.

Technical-industrial convergence: the candidate drug TNT005 represents a landmark case in which clinical trial entry was driven primarily by organ‑on‑a‑chip data. But the high costs of organ-on-a-chip platforms demand focused solutions through prioritization of emerging technologies. Additionally, there is a need to develop vascularized and neural-integrated multi-organ chips, as well as chips with stable evaluation capabilities and enhanced AI model generalization to better address clinical demands.

Maximize animal data value: researchers at the University of Zurich developed a technology that allows evaluating about 25 antibodies simultaneously in one mouse.^10^ Compared with traditional methods, it reduces the number of animals used in pharmacokinetic and biodistribution studies by up to 100 times. The approach of minimizing ethical issues without compromising data quality or violating regulatory standards is worth developing and utilizing.

We advocate for a balanced and progressive transition toward new approach methodologies. On the one hand, the unique information value of the long-accumulated animal testing data should be fully utilized; on the other hand, human-relevant NAMs should be verified through frameworks such as the FDA’s ISTAND. This paradigm shift presents both challenges and opportunities for enhancing translational efficiency and advancing the research ecosystem. We eagerly anticipate the emergence of more disruptive innovations with enhanced predictive accuracy, which will accelerate the drug development process, ultimately promoting human health and well-being.

## Funding and acknowledgments

This work was supported by the Zhejiang Provincial Key Research and Development Program (no. 2024C03003 to B.Y.), the 10.13039/501100002858China Postdoctoral Science Foundation (no. 2024M762888 to H.Z.), and the 10.13039/501100001809National Natural Science Foundation of China (no. 82504856 to H.Z.).

## Declaration of interests

The authors declare no competing interests.
